# Incidence of and factors associated with brimonidine allergy

**DOI:** 10.1371/journal.pone.0325319

**Published:** 2025-06-02

**Authors:** Sunee Chansangpetch, Pattawee Pongpisitkul, Kongkiart Tipparut, Kitiya Ratanawongphaibul, Rath Itthipanichpong, Anita Manassakorn, Visanee Tantisevi, Prin Rojanapongpun

**Affiliations:** 1 Department of Ophthalmology, Faculty of Medicine, Chulalongkorn University and King Chulalongkorn Memorial Hospital, Thai Red Cross Society, Bangkok, Thailand; 2 Center of Excellence in Glaucoma, Chulalongkorn University, Bangkok, Thailand; 3 Faculty of Medicine, Chulalongkorn University, Bangkok, Thailand; Kalinga Institute of Medical Sciences, INDIA

## Abstract

**Purpose:**

This study examined the incidence of and the factors associated with brimonidine allergy, as well as its clinical characteristics and management strategies.

**Methods:**

We conducted a retrospective review of brimonidine prescriptions and the medical charts of patients who were administered brimonidine between 2019 and 2020. The participants were divided into two groups according to the presence or absence of brimonidine allergy. Data on the demographic and clinical variables were collected for comparative analyses between the two groups.

**Results:**

A total of 12,024 brimonidine prescriptions were administered to 2,850 patients. Brimonidine allergy’s incidence was 5.5% (157 out of 2,850 patients). The median time from usage to the onset of allergic signs and symptoms was 32 weeks (interquartile range, 15–72 weeks). Conditional multivariable logistic regression analysis showed that brimonidine allergy was associated with concurrent topical steroid use (odds ratio [OR] = 0.18; 95% confidence interval [CI], 0.04 to 0.87; p = 0.033), concurrent artificial tear use (OR = 3.07, 95%, CI, 1.36 to 6.93; p = 0.007), and concurrent tafluprost use (OR = 3.63, 95% CI, 1.04 to 12.63; p = 0.043. The patients mostly experienced redness of the eyes (73.8%) and itching (50.0%). Most patients discontinued brimonidine usage (98.7%). The symptoms and signs improved after a median of 5.5 weeks of treatment.

**Conclusions:**

Approximately 5.5% of brimonidine users developed brimonidine allergy, which typically manifested at 32 weeks. Concurrent artificial tear and tafluprost use increased brimonidine allergy risk, whereas topical steroids’ concomitant use reduced the risk.

## Introduction

Glaucoma is the second leading cause of blindness. Several treatment options are available for glaucoma, including topical antiglaucoma medications, laser therapy, and surgery. Initial treatment usually includes topical antiglaucoma medications. These include alpha-2 adrenergic agonists, beta-adrenergic antagonists, carbonic anhydrase inhibitors, prostaglandin analogs, and parasympathomimetic agents.

Alpha-2 adrenergic agonists are commonly used to treat glaucoma. The drug reduces intraocular pressure (IOP) by decreasing aqueous humor production and increasing aqueous humor drainage through uveoscleral outflow. This drug is also used for neuroprotection [1,2].

Alpha-2 adrenergic agonists have been associated with a higher rate of ocular allergy than other antiglaucoma drugs. Apraclonidine, a drug in the alpha-2 adrenergic agonist group, is reported to have an allergy rate of up to 48%. [3] Brimonidine, another drug in this group, is a selective alpha-2 adrenergic agonist. It has a lower allergy rate than apraclonidine, ranging from 4.2% to 25.7%. [4–7] The symptoms and signs of brimonidine allergy, including itching, follicular/papillary conjunctivitis, and eyelid dermatitis, typically resolve after discontinuation of brimonidine.

Although allergy to brimonidine manifests locally, it has a negative influence on the patient’s quality of life. Restricting brimonidine use can hinder patients from achieving the target IOP. Reports from different countries have shown varying incidences of ocular allergies due to brimonidine treatment. [4–8] However, few studies have comprehensively examined the associated risk factors, clinical characteristics, and management strategies. Understanding these aspects is essential for early identification, timely intervention, and optimization of treatment in affected patients. This study aimed to evaluate the incidence of brimonidine allergy and the factors associated with it. In addition, the clinical characteristics of and management strategies for brimonidine allergy have been described.

## Materials and methods

This retrospective study was approved by the Institutional Review Board of Chulalongkorn University. The study was conducted in compliance with the tenets of the Declaration of Helsinki. We retrieved all prescription data containing brimonidine at the King Chulalongkorn Memorial Hospital, Thailand, between 1^st^ January 2019 and 31^st^ December 2020. The medical records of all the patients who were associated with these brimonidine prescriptions were subsequently reviewed. We excluded visits in which brimonidine was prescribed without an encounter note. The IRB granted a waiver for the requirement of informed patient consent. (IRB approval number 124/64)

Patients were classified as having an allergy to brimonidine if the diagnosis of “brimonidine allergy” was made by glaucoma specialists or verified by a pharmacist. Otherwise, the clinical information in the medical records was reviewed by an ophthalmologist (PP), and de-identified data were extracted. The patients were classified as having brimonidine allergy if they met all of the following criteria: 1) presence of itching, 2) follicular conjunctivitis/papillary conjunctivitis or eyelid dermatitis, and 3) improvement of these symptoms and signs after discontinuation of brimonidine.

Brimonidine users who did not have a brimonidine allergy were included in the study as 1:1 individual age- and sex-matched controls. For both allergic and control groups, the following information was collected: brimonidine usage (i.e., laterality of brimonidine use, concentration of brimonidine, brimonidine dosage, and duration of brimonidine use), concurrent medication use (i.e., antiglaucoma medications, topical steroids, and artificial tears), glaucoma status (i.e., diagnosis, previous glaucoma surgery, number of antiglaucoma drops, number of glaucoma drops with preservatives, best-corrected visual acuity, IOP, and cup-to-disc ratio), history of underlying allergic diseases, and history of drug allergies other than brimonidine. The data were obtained from the visit documenting a brimonidine allergy for the brimonidine-allergic group, and from the most recent visit with a brimonidine prescription for the non-allergic group. Additionally, data on the clinical course of and treatment for brimonidine allergy were obtained for eyes in the allergic group. The onset of allergy was defined as the time from the initiation of brimonidine to the first clinical documentation of a brimonidine allergy. If brimonidine was used in both eyes, one eye was selected by simple randomization (coin toss).

### Statistical analysis

Descriptive statistics were presented as means, standard deviations, medians, and interquartile ranges for continuous variables. Categorical variables were expressed as frequencies and percentages. The independent t-test or Mann–Whitney U test was used to compare continuous variables. Categorical variables were compared using the chi-squared or Fisher’s exact test. To determine the factors associated with brimonidine allergy, a subsequent conditional multivariable logistic regression analysis was carried out for parameters that showed p-values < 0.1 in the univariable analysis. The analysis was adjusted to account for the duration of brimonidine use. The significance level was set at p < 0.05. Statistical analysis was conducted using Stata 16.0 (StataCorp, College Station, TX, USA).

## Results

Between 2019 and 2020, 12,024 brimonidine prescriptions were administered to a total of 2,850 patients. Allergy to brimonidine was observed in 157 patients. Thus, the incidence of allergy to brimonidine was 5.5% (95% confidence interval [CI] 4.70–6.41).

**Fig 1:**
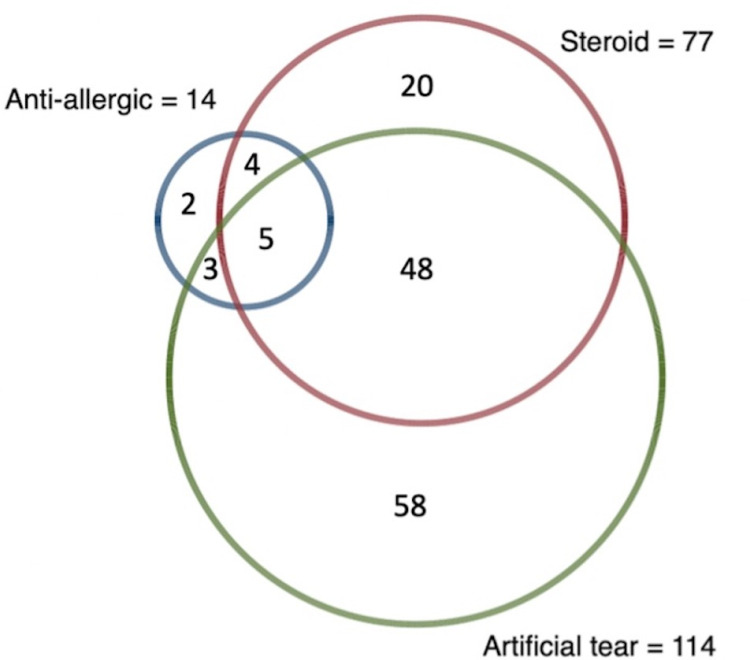
Venn diagram showing the prescribed medications for the management of brimonidine allergy.

The clinical characteristics of brimonidine allergy are summarized in **Table 1**. Patients usually reported at least one ocular symptom (80.3%). The most frequent symptoms were red eyes (73.8%) and itching (50.0%). Ocular signs were documented in 73.2% of the patients, the majority of which were follicular conjunctivitis (95.7%). The median time from usage to the onset of allergic signs and symptoms was 32 weeks, with an interquartile range of 15 to 72 weeks. Brimonidine was discontinued in 155 (98.7%) patients. The most frequently used medical treatments included topical steroids (49.0%), artificial tears (72.6%), and topical antiallergic drugs (8.9%). The symptoms and signs improved after a median of 5.5 weeks of treatment, with an interquartile range of 4–12 weeks. **Fig 1** shows the medications administered to treat the brimonidine allergy. The types of topical steroids used for treatment are shown in S1 Table.

**Table 1:** Clinical characteristics of brimonidine allergy.

Clinical characteristics	Values	
n	%
Presence of symptoms (n = 157)		
- Yes	126	80.3%
- No	5	3.2%
- Undocumented	26	16.6%
Symptoms (n = 126)		
- Itching	63	50.0%
- Red eye	93	73.8%
- Tearing	11	8.7%
- Lid swelling	19	15.1%
- Eye discomfort	5	4.0%
- Periorbital rash	1	0.8%
Signs (n = 157)		
- Yes	115	73.2%
- Undocumented	42	26.8%
Signs (n = 115)		
- Follicular conjunctivitis	110	95.7%
- Papillary conjunctivitis	10	8.7%
- Periorbital dermatitis	11	9.6%
- Non-specific conjunctival injection	1	0.9%
- Chemosis	1	0.9%

One hundred fifty-seven age- and sex-matched controls were included. The median duration of brimonidine usage for the non-allergic group was 100 weeks, with an interquartile range of 35 to 214 weeks. Table 2 shows the data on demographic and clinical variables in the brimonidine allergic and non-allergic groups. Brimonidine preparations of 0.15% and 0.2% were used in 78.0% and 22.0% of the patients, respectively, with no statistically significant differences in the usage rates of the two preparations between the groups. None of the patients in the non-allergy group had an underlying allergic disease, but seven patients in the brimonidine allergy group had an underlying allergic condition, including allergic rhinitis (3), asthma (3), allergic dermatitis (3), or a combination of these.

**Table 2:** Demographic and clinical variables in the brimonidine allergy and non-allergy groups.

Variables	Total (n = 314)	Brimonidine-Allergic (n = 157)	Non-Allergic (n = 157)	p-value
Age (years)	66.44 ± 9.87	66.36 ± 9.94	66.52 ± 9.84	0.867
Gender				1.000
- Male	174 (55.4)	87 (55.4)	87 (55.4)	
- Female	140 (44.6)	70 (44.6)	70 (44.6)	
Laterality of brimonidine use				0.118
- Right eye	84 (26.8)	43 (27.4)	41 (26.1)	
- Left eye	71 (22.6)	28 (17.8)	43 (27.4)	
- Both eyes	159 (50.6)	86 (54.8)	73 (46.5)	
Study eye				0.310
- Right eye	161 (51.3)	85 (54.1)	76 (48.4)	
- Left eye	153 (48.7)	72 (45.9)	81 (51.6)	
Concentration of brimonidine				0.785
- 0.15%	245 (78.0)	124 (79.0)	121 (77.1)	
- 0.2%	69 (22.0)	33 (21.0)	36 (22.9)	
Brimonidine dosage				<0.001
- 2 times a day	221 (70.4)	125 (79.6)	96 (61.1)	
- 3 times a day	93 (29.6)	32 (20.4)	61 (38.9)	
History of allergic diseases	7 (2.2)	7 (4.5)	0 (0)	0.015 ^a^
History of drug allergy				0.001
- None	229 (72.9)	105 (66.9)	124 (79.0)	
- Oral medication	70 (22.3)	38 (24.2)	32 (20.4)	
- Topical antiglaucoma medication	15 (4.8)	14 (8.9)	1 (0.6)	
Diagnosis				0.224 ^a^
- Primary glaucoma	269 (85.7)	139 (88.5)	130 (82.8)	
- Secondary glaucoma	39 (12.4)	14 (8.9)	25 (15.9)	
- Childhood glaucoma	3 (1.0)	2 (1.3)	1 (0.6)	
- Nonglaucoma optic neuropathy	3 (1.0)	2 (1.3)	1 (0.6)	
Previous glaucoma surgery				0.485 ^a^
- None	272 (86.6)	132 (84.1)	140 (89.2)	
- Trabeculectomy	37 (11.8)	21 (13.4)	16 (10.2)	
- Glaucoma drainage device	3 (1.0)	2 (1.3)	1 (0.6)	
- Trabeculotomy	1 (0.3)	1 (0.6)	0 (0)	
- Deep sclerectomy	1 (0.3)	1 (0.6)	0 (0)	
Concurrent topical steroid	32 (10.2)	8 (5.1)	24 (15.3)	0.003
Concurrent artificial tear	222 (70.7)	118 (75.2)	104 (66.2)	0.083
Concurrent prostaglandin				0.044 ^a^
- None	125 (39.8)	63 (40.1)	62 (39.5)	
- Latanoprost	129 (68.3)	64 (68.1)	65 (68.4)	
- Bimatoprost	19 (10.1)	5 (5.3)	14 (14.7)	
- Travoprost	9 (4.8)	3 (3.2)	6 (6.3)	
- Tafluprost	32 (16.9)	22 (23.4)	10 (10.5)	
Concurrent beta-blocker				0.032 ^a^
- None	177 (56.4)	97 (61.8)	80 (51.0)	
- Timolol	133 (42.4)	58 (36.9)	75 (47.8)	
- Carteolol	2 (0.6)	2 (1.3)	0 (0)	
- Betaxolol	2 (0.6)	0 (0)	2 (1.3)	
Concurrent carbonic anhydrase inhibitor				0.355
- None	205 (65.3)	104 (66.2)	101 (64.3)	
- Brinzolamide	19 (6.1)	12 (7.6)	7 (4.5)	
- Dorzolamide	90 (28.7)	41 (26.1)	49 (31.2)	
- Concurrent pilocarpine	0 (0)	0 (0)	0 (0)	
- Number of antiglaucoma drops	3 (2-5)	3 (2-5)	4 (2-5)	0.047
- Use of systemic antiglaucoma	13 (4.1)	3 (1.9)	10 (6.4)	0.047 ^a^
Visual acuity (logMAR)	0.2 (0.1-0.5)	0.1 (0.0-0.33)	0.3 (0.1-0.75)	0.002
Intraocular pressure (mmHg)	14 (11-17)	14 (11.5-18)	14 (11-17)	0.427
Cup to disc ratio	0.8 (0.6-0.9)	0.8 (0.6-0.9)	0.8 (0.6-1.0)	0.429

Data shown in mean ± standard deviation, median (interquartile range), or n (%).

P values were calculated by the independent t-test, Mann-Whitney U test, and Chi-square test for data presented with mean, median, and count, respectively. ^a^ Fisher’s exact test

From the univariable analysis, the following variables showed a p-value of less than 0.1: brimonidine dosage, history of allergic diseases, history of drug allergy, concurrent use of topical steroids, artificial tears, prostaglandins, beta-blockers, number of antiglaucoma drops, use of systemic antiglaucoma medications, and visual acuity. All variables—except for history of allergic diseases, which had no events in the control group—were subsequently included in the conditional multivariable logistic regression analysis. The results of the multivariable analysis are shown in Table 3. A history of allergy to topical antiglaucoma medication was found to increase the likelihood of allergy to brimonidine with an odds ratio (OR) of 463.45 (95% CI, 10.43–20.6 × 10^3^, p = 0.002). A higher risk of brimonidine allergy was also found in the patients who concurrently used artificial tears, with an OR of 3.07 (95% CI, 1.36–6.93, p = 0.007), and tafluprost, with an OR of 3.63 (95% CI, 1.04–12.63, p = 0.043). However, concurrent steroid use reduced the risk of brimonidine allergy with the OR of 0.18 (95% CI, 0.04–0.87, p = 0.033).

**Table 3:** Conditional multivariable logistic regression analysis for variables associated with brimonidine allergy.

	Odds Ratio	95% confidence interval		
		Lower	Upper	p-value
Visual acuity (logMAR)	1.03	0.61	1.77	0.897
History of drug allergy				
- None	reference			
- Oral medication	1.51	0.69	3.32	0.297
- Topical antiglaucoma medication	463.45	10.43	20.6 x 10^3^	0.002
Concurrent topical steroid (Yes)	0.18	0.04	0.87	0.033
Concurrent artificial tear (Yes)	3.07	1.36	6.93	0.007
Brimonidine dosage (Three times a day) ^#^	0.52	0.19	1.42	0.201
Concurrent prostaglandin				
- None	reference			
- Latanoprost	2.16	0.89	5.28	0.090
- Bimatoprost	0.41	0.09	1.79	0.234
- Travoprost	0.31	0.02	4.33	0.381
- Tafluprost	3.63	1.04	12.63	0.043
Concurrent beta-blocker (Yes)	0.67	0.23	1.97	0.464
Number of antiglaucoma drops	0.95	0.65	1.41	0.810
Use of systemic antiglaucoma (Yes)	0.24	0.03	1.78	0.162

Adjusted for duration of brimonidine use.

# Reference to twice a day dosage.

## Discussion

Brimonidine is a commonly prescribed antiglaucoma medication. Brimonidine allergy affects not only the patients’ quality of life but also their treatment outcomes. The incidence of ocular allergy to brimonidine at the King Chulalongkorn Memorial Hospital from 2019 to 2020 was 5.5%. The median time from the usage of medication to appearance of allergies was 32 weeks. A history of allergy to topical antiglaucoma medication, concurrent topical steroid use, concurrent artificial tear use, and concurrent tafluprost use were associated with brimonidine allergy.

The 5.5% incidence rate in our study was consistent with the rates reported in prior studies, i.e., 4.2% to 25.7%. [4–7] Notably, each study defined the allergy rate slightly differently. Some studies, including ours, included all brimonidine users, both current and newly prescribed. [12] Other studies, on the other hand, specifically explored the allergy rate among new brimonidine users. [4–7] Most patients with brimonidine allergy experienced red eyes and itching. Follicular conjunctivitis was also frequently observed. The three most commonly used treatments, in order of frequency, were artificial tears alone, topical steroids combined with artificial tears, and topical steroids alone. Brimonidine was discontinued for almost all affected patients. Seventeen patients showed complete resolution after discontinuation of brimonidine without receiving any additional treatment. Two patients were able to continue using brimonidine because they reported no symptoms and had only minor allergic reactions. Therefore, brimonidine can be prescribed for patients who can tolerate allergic reactions. However, the treatment should be discussed with these patients, and the patients should be monitored during follow-up.

Our findings showed an onset of brimonidine allergy at 32 weeks, which is consistent with prior reports indicating an onset ranging from 7–12 months. [8–10] It has been speculated that the allergic response may be related to drug metabolites that become immunogenic through interactions with cellular macromolecule, forming haptens that subsequently sensitize T cells. Because of this process, the onset of allergy may be more delayed response than what is typically observed from classic delayed type hypersensitivity reactions [11].

Our study showed that concurrent use of topical steroids was negatively associated with brimonidine allergy. Topical steroids are one of the therapeutic agents used for treating allergic conjunctivitis. [12] Since steroids suppress allergic reactions by reducing the levels of inflammatory cytokines, patients who used topical steroids concurrently were less likely to develop symptoms and signs of brimonidine allergy.

The patients who concurrently used artificial tears and brimonidine were more likely to be allergic to brimonidine. A previous study reported an association between brimonidine allergy and reduction in tear film production. [6] The need for artificial tears indicated that these patients had underlying ocular surface problems such as dry eye syndrome, which may facilitate the expression of ocular allergic reactions due to reduced surface clearance and lead to decreasing allergens and inflammatory mediators clearance, [13] resulting in changes in the epithelial barrier [14].

Follicles are typically not observed in allergic reactions of the conjunctiva, but they may be a key indicator of certain drug toxicity. The majority of the chemicals listed as causatives of allergies are halogenated compounds, including brimonidine, demecarium bromide, idoxuridine, furtrethonium iodide, trifluridine, diisopropylfluorophosphate, and echothiophate iodide. [15] Our results showed that concurrent tafluprost use increased the risk of brimonidine allergy. Tafluprost is a prostaglandin analog with two fluorine atoms replacing the C-15 hydroxyl group, unlike other prostaglandin analogs. Fluorine, which is found in tafluprost, and bromine, which is found in brimonidine, are halogens. Halogenated compounds occasionally cause allergic reactions in the skin known as halogenoma. The term halogenoma encompasses the rare conditions of bromoderma, iododerma, and fluoroderma, characterized by skin lesions related to bromide, iodide, and fluoride exposure, respectively. [16] The correlation between the use of these halides and conjunctival follicular reaction is currently unknown. We speculate that the concurrent use of two halogenated eye drops may increase the risk of allergy in susceptible patients. Notably, travoprost also contains fluorine atoms, although its chemical structure and location differ from those of tafluprost. Interestingly, follicular conjunctivitis has also been reported as a complication of both tafluprost and travoprost; however, this is rare. [17,18] This study included very few patients who used travoprost; hence, the relationship between travoprost and brimonidine allergy could not be elucidated. Another alternative explanation for the increased risk of brimonidine allergy among tafluprost users is that the tafluprost used in our clinic was a single-unit, non-preservative formula. Thus, it was frequently prescribed to patients with ocular surface diseases, including dry eye syndrome, which is a risk factor as mentioned earlier. Further research on the potential relationship between tafluprost use and brimonidine allergy and its relationship to halogenated elements is required.

Both a history of allergic diseases and allergy to topical antiglaucoma medication were found to be associated with brimonidine allergy in our study. The brimonidine group included a significantly higher proportion of patients with a history of allergic diseases than the control group. Among the patients with brimonidine allergy, 4.5% had a history of allergic diseases such as allergic rhinitis, asthma, and allergic dermatitis. Previous studies have shown an association between atopy and allergy to oral medications such as amoxicillin [19] and nonsteroidal anti-inflammatory drugs. [20] However, no study has reported the association between atopy and allergy to ocular medications. Some studies have shown that while patients with atopy do not appear to be predisposed, [21] their allergic reactions can be more serious. Although the association was suggested in the univariable analysis, we were unable to incorporate this factor into the multivariable logistic regression analysis due to the absence of cases in the non-allergic group. A history of allergy to topical antiglaucoma medication was also identified as a risk factor for brimonidine allergy in previous studies. [4,6] This may be explained by the tendency to show hypersensitivity responses in these patients. Notably, the small number of participants with a history of topical antiglaucoma medication (14 in the allergic group and 1 in the non-allergic group) resulted in a high OR and wide 95% CI. Further research involving larger study populations with a history of antiglaucoma medication is warranted to yield a more precise risk estimation for this factor.

Concomitant levobunolol use has previously been shown to be associated with a high risk of brimonidine allergy [4]. The authors speculated that both brimonidine and levobunolol contain either polyvinyl alcohol or haptens, and simultaneous usage of both medications crossed the threshold for allergic induction. However, our study showed that concurrent beta-blocker use was more likely to reduce allergy, although this effect was not statistically significant in the multivariable model. Beta-blockers may have a vasoconstrictive effect, decreasing inflammation, [22] and may block conjunctival cell volume reduction, lower intercellular fluid flow, and limit proinflammatory mediator access. [23] In addition, none of the patients in our study used levobunolol.

The strength of our study is that it assessed the largest number of patients with brimonidine allergy to date. However, our study had some limitations. First, the retrospective nature of the study resulted in problems associated with incomplete medical records. In addition, the visit notes tended to mention only positive findings; therefore, for undocumented findings, we were unable to differentiate between negative findings and findings that were not assessed. Thus, some clinical characteristics may have been underreported. Second, the sample size was insufficient to adequately characterize certain factors associated with brimonidine allergy, such as a history of allergic disease. Third, there is currently no consensus regarding the diagnostic criteria for brimonidine allergy; thus, the diagnoses made by ophthalmologists may have been subjective and variable. However, we classified brimonidine allergies based on the judgment of glaucoma specialists to minimize variation. We also consistently used criteria that required improvement of symptoms and signs after discontinuation of brimonidine to ensure that the allergic reaction was not caused by other concomitant medications or conditions. Forth, 0.1% brimonidine was not available at our hospital during the study period, limiting the generalizability of the results to this newly released concentration. Finally, we were unable to determine whether the patients were allergic specifically to brimonidine or other chemical compounds in brimonidine medications.

In conclusion, approximately 5.5% of the brimonidine users developed brimonidine allergy, which typically manifested at 32 weeks. Concurrent artificial tear use, concurrent tafluprost use, and allergy to other topical antiglaucoma medications increased the risk of brimonidine allergy, whereas concomitant use of topical steroids reduced the risk. Proper advice and monitoring should be provided to patients who are at risk of brimonidine allergy.

## Supporting information

S1 File Dataset. (XLSX)

S1 Table Type of topical steroids used for treating brimonidine allergy. (DOCX)
