# Supplementary material for: Incidence of and factors associated with brimonidine allergy
Source: PLoS One. 2025 Jun 2;20(6):e0325319. doi: 10.1371/journal.pone.0325319 (PMC12129204; doi:10.1371/journal.pone.0325319)
Supplement: S1 Table — (DOCX) [file pone.0325319.s002.docx]

**Table, Supplemental Digital Content 1.** Type of topical steroids used for treating brimonidine allergy

| Steroid treatment (n=157) | N | % |
| --- | --- | --- |
| no | 80 | 51.0% |
| yes | 77 | 49.0% |
| Fluorometholone | 30 | 19.1% |
| Prednisolone acetate | 3 | 1.9% |
| 0.5% Loteprednol | 22 | 14.0% |
| 0.2% Loteprednol | 2 | 1.3% |
| Dexamethasone | 7 | 4.5% |
| Methylprednisolone | 12 | 7.6% |
| 0.1% Triamcinolone cream | 1 | 0.6% |
